# Identification of a novel SARS-CoV-2 variant with a truncated protein in ORF8 gene by next generation sequencing

**DOI:** 10.1038/s41598-022-08780-2

**Published:** 2022-03-17

**Authors:** Stephanie DeRonde, Hannah Deuling, Jayme Parker, Jack Chen

**Affiliations:** 1 Department of Biology and Wildlife, Institute of Arctic Biology, University of Alaska Fairbanks, Fairbanks, AK 99775 USA; 2 Alaska State Virology Laboratory, Fairbanks, AK 99775 USA

**Keywords:** Infectious-disease diagnostics, Virology

## Abstract

Using next generation sequencing technology, we identified a novel SARS-CoV-2 variant with a truncated ORF8 protein mutation near the end of the viral genome from nucleotides 27,878 to 27,958. This point mutation from C to T at nucleotide 27,956 changed the amino acid codon CAA (glutamine) to a stop codon, TAA, created a novel stop codon in ORF8 gene, resulting in a much smaller ORF8 protein (26 aa) than the wild type ORF8 protein (121 aa). This variant belongs to Pango lineage B.1.1291, which also contains the D614G mutation in the Spike (S) gene. The B.1.1291 lineage is predominantly circulated in the United States of America (97.18%), although it was also found in other counties (Russia, Canada, Latvia, Chile, India, Japan, Colombia, Germany, Greece, Mexico, and UK). A total of 340 closely related variants to this novel variant were identified in GISAID database with collection dates ranged from 3/6/2020 to 10/21/2020. In addition, a search within NCBI Genbank database found that 108,405 of 873,230 (12.4%) SAR-CoV-2 complete genomes contain this truncated ORF8 protein mutation, indicating this mutation may arise spontaneously in other lineages as well. The wide distribution of this mutation indicates that this truncated ORF8 protein mutation may provide the virus a growth advantage and adaptive evolution.

## Introduction

The severe acute respiratory syndrome coronavirus 2 (SARS-CoV-2) is responsible for the ongoing global pandemic of COVID-19. Understanding the molecular epidemiology and ongoing evolution of SARS-CoV-2 will provide critical insights for preparing for and preventing future pandemic. As an RNA virus, the SARS-CoV-2 genome shows high mutation rate and undergoes the impact of natural selection. The spike (S) protein of SARS-CoV-2, which enables the virus to binds the cellular receptor, ACE-2 and enter host cells, exhibits exceptional high mutations, leading to an effective S protein capable of infecting human cells more efficiently, and is the key force for the emerging of novel variants such as, Alpha, Delta and Omicron. The global spread and explosive growth of the SARS-CoV-2 infected population have contributed additional mutational variability into the viral genome. SARS-CoV-2 was first identified in Wuhan, China in December 2019 as the cause of COVID-19 disease. Since then, the virus has spread to a global level causing 57,882,183 confirmed COVID-19 infections and 1,377,395 deaths as of November 22, 2020^1^. Symptoms of COVID-19 include fever, cough, shortness of breath and difficulty breathing among other symptoms. Severe cases of COVID-19 can result in respiratory disease and pneumonia requiring hospitalization^2^. It was declared a global pandemic in March 2020 by the World Health Organization and since has caused a negative effect on the health, socio-economics and the lives of billions of people^3^.

SARS-CoV-2 is one of seven coronaviruses found to infect humans. It is an enveloped virus of single stranded RNA that codes for 6 major open reading frames (ORFs); ORF1a, ORF1b, spike (S), envelope (E), membrane (M) and nucleocapsid (N) among a few smaller ORF^4^. The genome sequence of SARS-CoV-2 was published early during COVID-19 pandemic, which shows that it is most closely related to a bat coronavirus—RaTG13^5^. This led researchers to believe that it most likely got transferred from a bat through an unknown intermediate source to humans^6^. SARS-CoV-2 has spread further and impacted more people than any other coronavirus. Knowing its genome structure is important for understanding why it has such a high transmission ability compared to other coronaviruses.

Alaska State Virology Laboratory began to sequence SARS-CoV-2 in March 2020 when the first case reached Alaska, shortly after the first case of COVID-19 was identified in US. We have been working on sequencing positive COVID-19 specimens collected from across Alaska. We are sequencing the SARS-CoV-2 genome using Next Generation Sequencing technology in order to get the full genome coverage of the virus in each sample. Understanding the genomic variation of this virus is key for tracing the spread of the virus. Genomic sequencing is also important in monitoring how the virus variants are circulating in local population, which provides first-hand information to health administration for prevention and intervention as well as sequencing data for vaccine development.

SARS-CoV-2 carries the largest single-stranded RNA genome. The 29,903-nucleotide genome of SARS-CoV-2 is composed of 13–15 (12 functional) open reading frames (ORFs) including a pp1ab polyprotein, four structural proteins and six accessory proteins; 3a, 6, 7a, 7b, 8, and 10^7^. ORF8 is an accessory protein that is still being understood with its function in regard to the infectability of SARS-CoV-2. A previous report shows that ORF8 protein may affect immune response in humans^8^. During sequencing of positive COVID-19 specimens, we found a novel SARS-CoV-2 variant with a truncated mutation in the ORF8 gene. Mutations in the ORF8 protein may affect the viral infectivity and provides the viral mutant the growth advantage over the wild type. Understanding if this is a random mutation or a case of adaptive evolution of the virus is crucial when looking at creating a productive vaccine. One study found that one of the functions of the ORF8 gene could be coding proteins that are associated with viral replication. This could mean a truncated mutation may decrease replication in the host and affect invasion of host immune response. Another potential function of ORF8 is immune modulation through hijacking the host ubiquitin proteasome system (UPS)^8^. This could help the virus get through the host immune surveillance system and start proliferating inside humans. Overall, we need further research into the functional properties of the ORF8 protein and how it could be affected when truncated.

## Materials and methods

### Clinical specimens

Positive COVID-19 samples were collected in Alaska State Virology Laboratory of Alaska State Public Health Laboratories from testing centers around the State and frozen in − 80 °C freezers until ready for RNA extraction and real time PCR testing. COVID-19 positive specimens were further subjected for genomic sequencing. This research project was reviewed and approved by the University of Alaska Fairbanks Institutional Review Board (IRB) (Approval letter No. 667418–4). All methods were performed in accordance with the relevant CDC guidelines and regulations.

### Bio-containment

All COVID-19 samples were tested in Alaska State Virology Laboratory (ASVL), a state public health laboratory with CLIA certification as a high complexity clinical laboratory. Samples in Viral Transport Media (VTMs) were extracted in ASVL before testing and positive samples (RNAs) were used in this study.

### Protocols and sequencing library prep kit

The protocols used to construct sequencing libraries was modified from instruction of NEBNext Ultra II RNA Library Prep Kit for Illumina (New England Biolabs, Cat. No. NEB #E7770).

### Construction of next generation sequencing library for illumina sequencer MiSeq

RNA fragmentation and priming were completed by adding 13ul of each sample RNA to primers and loading in a thermal cycler. Samples underwent first strand cDNA synthesis by adding NEBNext First Strand Synthesis enzyme mix and then second strand cDNA synthesis through adding buffer and Second Strand Synthesis enzyme mix in thermal cycler. To purify the double-stranded cDNA, 144ul of AMPure XP beads were added to second strand cDNA synthesis and transferred to magnetic rack. After 3 min the supernatant was discarded without disturbing the beads that contained the DNA target. 80% ethanol was added and removed to remove residue and clean the beads. The beads were left for 3 min for air dry instead of the protocol’s suggested 5 min because the reaction was performed in Fairbanks where the air is drier than most places. Next, 0.1X TE buffer was added to the dried beads then 50 μl of the supernatant was removed and transferred to a clean nuclease-free PCR tube. Some samples were stored at − 20 °C overnight at this point.

To complete end repair/dA-tail of cDNA library, NEBNext Ultra II End Repair Reaction buffer and enzyme mix were added to cDNA and incubated in thermal cycler. To perform adaptor ligation, NEBNext Ligation Enhancer, Adaptor (15 μM) and Blunt/TA Ligase Master Mix were added to samples while in thermal cycler. To purify the ligation reaction, AMPure XP beads were added and put on a magnetic rack. The same process as previously to clean the beads was performed. When done, 15 μl of the supernatant was transferred to a clean PCR tube to complete PCR enrichment of adaptor ligated DNA. To do this, NEBNext Ultra II Q5 Master Mix and Universal PCR Primer (10 μM) were both added to samples and put in a thermal cycler.

Next, AMPure XP Beads were added to purify the PCR reaction. The same process as previously to clean the beads was performed and then 20 μl of the supernatant was transferred to a clean 1.5 ml tube. To pool NGS library, 5 μl of each individual NGS library was taken and pooled into one 1.5 ml tube. 0.8X volume of resuspended Agencourt AMPure XP Beads were added to the tube and then the same process as previously to clean the beads was performed. At the end, 20 μl of the supernatant was transferred to a clean 1.5 ml tube.

### Quality control of sequencing library

To assess library quality, final NGS Library Pool was analyzed using Thermo Fisher Scientific Qubit Fluorometer and Agilent Bioanalyzer with Agilent DNA 1000 Kit. Based on results from Qubit and Agilent Bioanalyzer, the final NGS Library Pool concentration was diluted to 4 nM. For the final step and to prepare sequencing library, 4.5 μL of pure H_2_O, 0.5 μL of the 2 N NaOH, 4.5 μL of the 4 nM final NGS Library Pool and 0.5 μL of the 4 nM PhiX were all added to PCR tube and put in thermal cycler. Finally, 10 μL of the denatured library was transferred into the 990 μL HT1 buffer, resulting in 20 pM denatured Library/PhiX with 10% PhiX library.

### Sequencing run at Illumina Miseq system

Illumina MiSeq Reagent Kit v2 (2 × 250 bp) (Cat. No. MS-102–2003) was used in this study. 600 μL of the final NGS Library Pool containing 10% PhiX library were loaded on the cartridge to start sequencing run. Output using MiSeq Reagent Kit v2 (2 × 250 bp) is predicted to yield a total of 24–30 million sequencing reads, equal to 7.5–8.5 G bp.

### Bioinformatic analysis

Raw sequencing data were trimmed from adaptor and barcode sequencing using integrated software within Illumina Miseq system. Sequencher and QIAGEN CLC Genomics Workbench were used for genome assembly. The original SARS-CoV-2 Wuhan-Hu-1 genome sequence (NCBI Reference Sequence: NC_045512.2) was used as the reference genome for assembly. Full genome sequence was submitted to GISAID and NCBI Genbank. CoViZu, an open source COVID-19 genome analysis tool provided by the GISAID Initiative was used to visualize the global diversity of SARS-CoV-2 genomes an for sequencing and phylogenetic analysis.

### Ethical approval

This research project was reviewed and approved by the University of Alaska Fairbanks Institutional Review Board (IRB) (Approval letter No. 667418-4). All methods were performed in accordance with the relevant guidelines and regulations.

## Results

**Figure 1:**
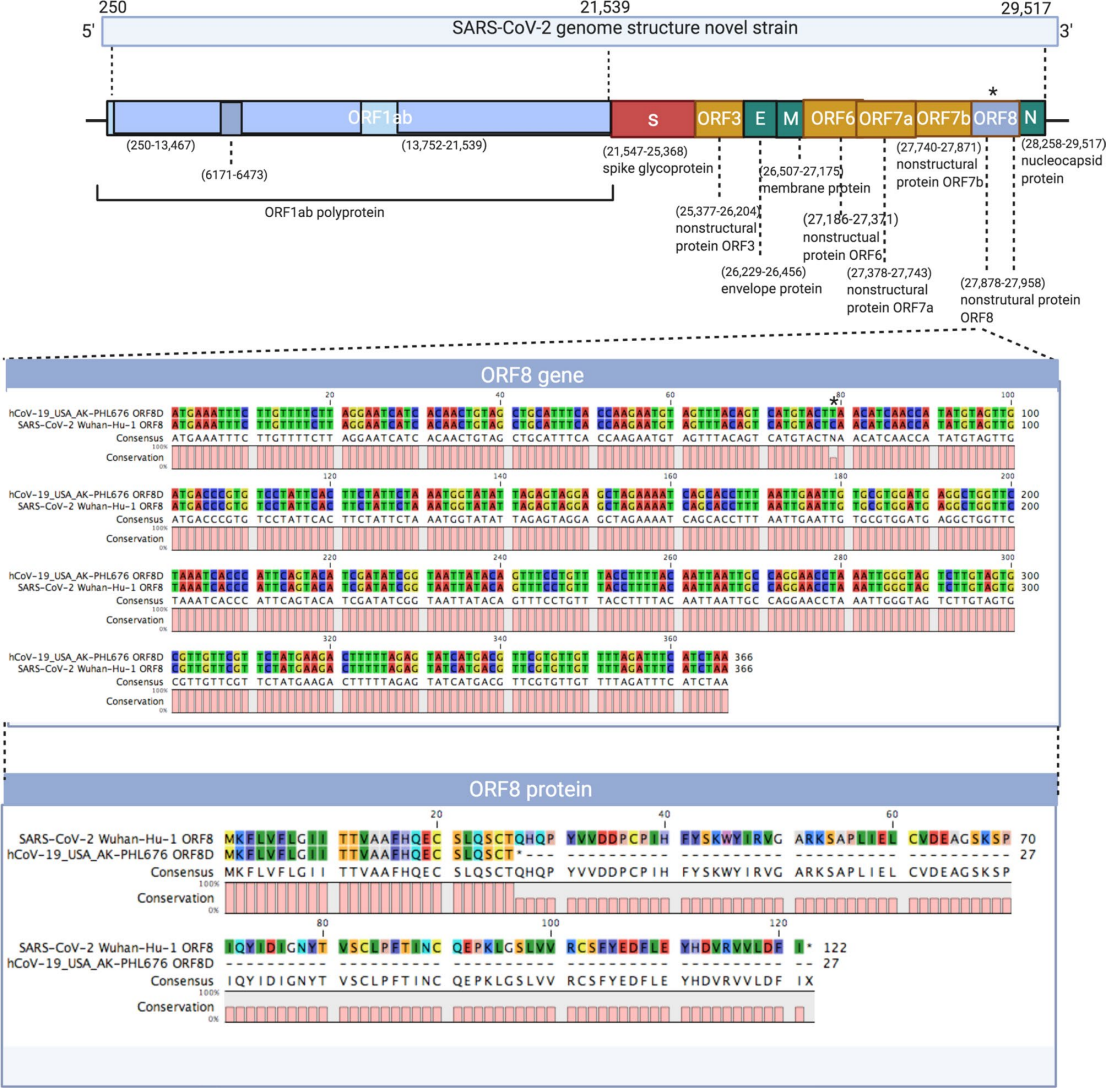
Genomic structure of novel SARS-CoV-2 variant. ORF8 gene sequences is enlarged and shown along with ORF8 amino acid sequences for both SARS-CoV-2 Wuhan-Hu-1 reference sequence and hCoV-19/USA/AK-PHL676/2020 truncated mutation variant. The reference sequence SARS-CoV-2 Wuhan-Hu-1 shows the wild type gene length which translates to the ORF8 protein. * indicates where the point mutation occurred creating the truncation in the ORF8 protein. This figure was created using CLC Sequencing Viewer, a part of CLC Genomics Workbench, for alignment of multiple sequences. Consensus sequence is determined by the software, it gives an identical nucleotide when all sequences at that position are the same, and a different nucleotide (in this case, a N) to represent the difference in each sequence. Conservation is also determined by the software, and is presented as 0–100% conservation value in a specific position.

From our routine sequencing work using whole genomic RNA sequencing approach, we identified a novel SARS-CoV-2 variant with a truncated ORF8 protein mutation. We obtained a total sequencing reads of 2,639,262 from this sample, we used a bioinformatics software called Sequencher and QIAGEN CLC Genomics Workbench for SARS-CoV-2 genome assembly and used the original SARS-CoV-2 Wuhan-Hu-1 genome sequence (NCBI Reference Sequence: NC_045512.2) as the reference genome. A total of 2392 SARS-CoV-2 viral reads were identified from this sample, which was further assembled into a full viral genome, resulting in 20X sequencing depth. This sequencing depth is able to achieve viral genome coverage and discovering accurate variants, with greater than 99% genome coverage. As shown in Fig. 1, the truncated mutation is located in the ORF8 gene which is near the end of the genome from nucleotides 27,878 to 27,958. The normal ORF8 gene is from nucleotides 27,878 to 28,246. The wild type ORF8 protein is 121 aa. This truncation is caused by a point mutation from a C to a T in nucleotide position at 27,956, which changed the amino acid codon from CAA (glutamine) to a stop codon TAA (Fig. 1), resulting in a truncated, smaller ORF8 protein (26 aa), compared with wild type ORF8 protein (121 aa).

As shown in Table 1, this novel variant (hCoV-19/USA/AK-PHL676/2020) was assigned as Pango Lineage B.1.1.291 by GISAID Initiative (https://www.gisaid.org, Accession ID: EPI_ISL_586254). Comparison of this novel variant with genome signature of B.1.1.291 indicates that this novel variant contains two unique mutations (ORF8 Q27stop, NSP15 V127F) that other B.1.1.291 variants do not have, while lack of some other mutations (ORF1ab F106F, ORF1ab Y831Y, ORF1ab N73N, noncoding C29784T, and noncoding C241T) in Pango Lineage B.1.1.291 (Table 1). Interestingly, the only nonsynonymous mutation in this novel variant different from other Pango Lineage B.1.1.291 is the ORF8 Q27stop mutation, while all other differences are either synonymous mutations, or nonsynonymous mutations in non-coding region (Table 1).

**Table 1:** Comparison of hCoV-19/USA/AK-PHL676/2020 with genome signature of B.1.1.291.

Gene/ORF	Mutation site	Mutation type	B.1.1.291	hCoV-19/USA/AK-PHL676/2020
Spike gene	D614G	Non-syn	+	+
N gene	R203K	Non-syn	+	+
N gene	G204R	Non-syn	+	+
ORF8 gene	Q27stop	Non-syn	−	+
ORF1ab/nsp3	F106F	Syn	+	−
ORF1ab/nsp12	P323L	Non-syn	+	+
ORF1ab/nsp12	Y831Y	Syn	+	−
ORF1ab/nsp13	E244D	Non-syn	+	+
ORF1ab/nsp15A1-B	N73N	Syn	+	−
ORF1ab/nsp15A1-B	V127F	Syn	−	+
noncoding	C29784T	Non-syn	+	−
noncoding	C241T	Non-syn	+	−

The Pango Lineage B.1.1.291 was first identified in USA in March 9, 2020 (https://www.gisaid.org). So far, there are a total of 1347 virus isolates in Pango Lineage B.1.1.291 (Table 2). Most of them (1309 or 97.2%) were identified in the USA (Table 2). A search within NCBI Genbank database found that 108,405 of 873,230 (12.4%) SAR-CoV-2 complete genomes published so far contain truncated ORF8 protein mutations, indicating this mutation may arise spontaneously in other lineages as well.

**Table 2:** Distribution of the Pango Lineage B.1.1.291 in different countries.

Lineage	Country	Count	Percentage (%)	Earliest Epi week	Latest Epi week
B.1.1.291	All	1347	100.00	11	86
B.1.1.291	USA	1309	97.18	11	82
B.1.1.291	Russia	10	0.74	64	86
B.1.1.291	Canada	7	0.52	33	37
B.1.1.291	Latvia	7	0.52	61	66
B.1.1.291	Chile	3	0.22	55	56
B.1.1.291	India	3	0.22	41	51
B.1.1.291	Japan	2	0.15	31	73
B.1.1.291	Colombia	2	0.15	48	53
B.1.1.291	Germany	1	0.07	62	62
B.1.1.291	Greece	1	0.07	43	43
B.1.1.291	Mexico	1	0.07	77	77
B.1.1.291	UK	1	0.07	15	15

Epi Week: the week since COVID-19 pandemic.

**Figure 2:**
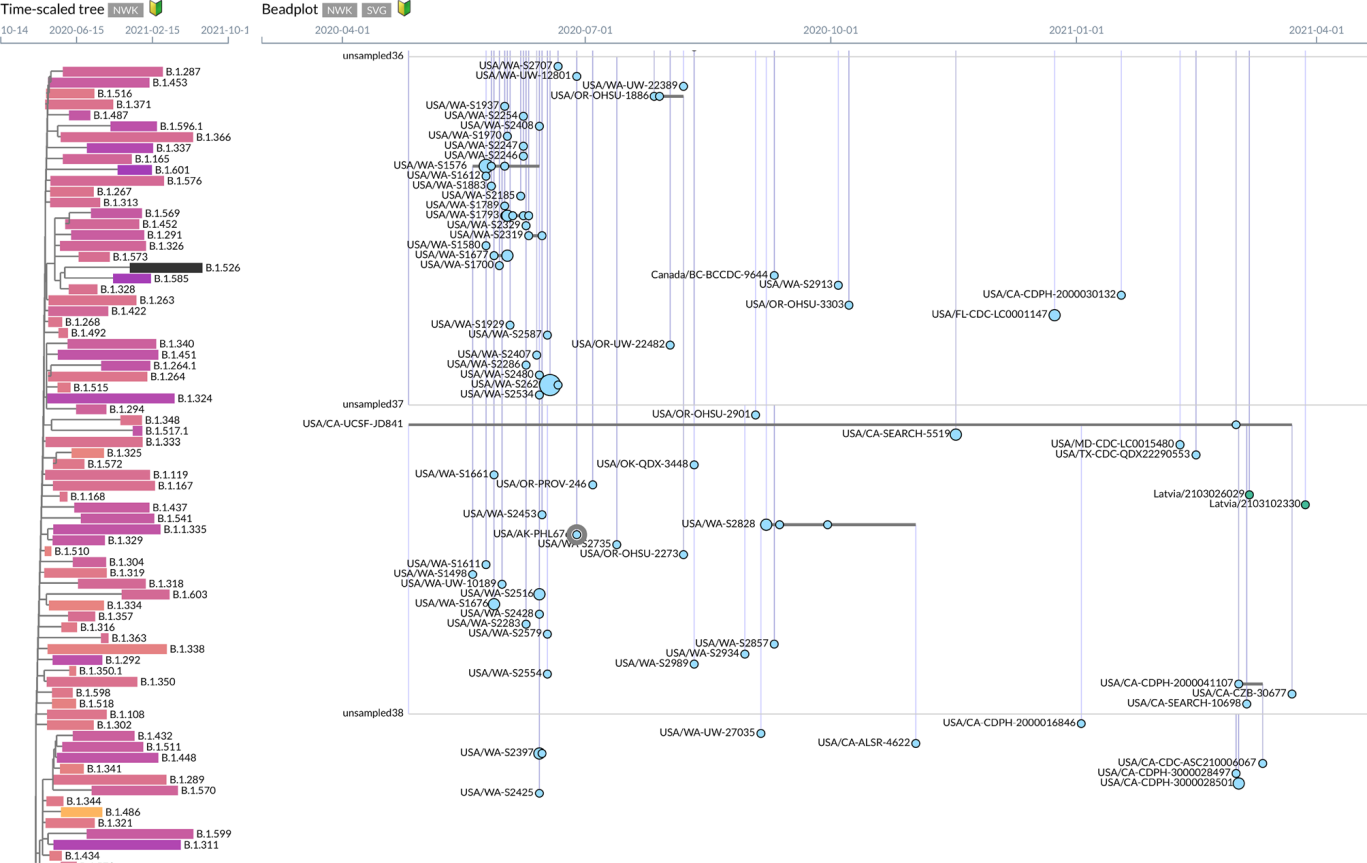
CoVizu analysis of visualizing the phylogenetic tree of most closely related variants with AK-PHL676 genome (highlighted). This analysis provides two visualizations of these data. On the left, it displays a phylogenetic tree summarizing the evolutionary relationships among different SARS-CoV-2 lineages^9^. It shows that AK-PHL676 belongs to linage B.1.526. On the right, it displays a “beadplot” visualization. Each horizontal line represents one or more samples of SARS-CoV-2 that share the same genome sequence. Beads along the line represent the dates that this variant was sampled. Vertical line segments were drawn to connects variants to their common ancestors. AK-PHL676 genome is related to unsample36.

CoVizu is an open-source analysis tool endeavoring to visualize the global diversity of SARS-CoV-2 genomes, which is provided by the GISAID Initiative. We used CoVizu analysis to visualize the variant we reported here—hCoV-19/USA/AK-PHL676/2020. The CoVizu analysis provides two interactive visualizations of these data. As shown in Fig. 2, on the left it displays a phylogenetic tree summarizing the evolutionary relationships among different SARS-CoV-2 lineages (groupings of viruses with similar genomes)^9^. hCoV-19/USA/AK-PHL676/2020 belongs to linage B.1.526. On the right of Fig. 2, the B.1.526 lineage displays a “beadplot” visualization. Each horizontal line segment represents a variant—viruses with identical genomes. Beads along a line indicate when that variant was sampled. If there are no beads on the line and it is grey, then it is an unsampled variant: two or more sampled variants descend from an ancestral variant that has not been directly observed. Beads along the line represent the dates that this variant was sampled. These beadplots can be used to visualize the different variants of SARS-CoV-2 within a lineage, where and when they have been sampled, and how they are related to each other. The area of the bead is also scaled in proportion to the number of times the variant was sampled that day. Vertical line segments are used to connect variants to their common ancestors. As shown in Fig. 2, hCoV-19/USA/AK-PHL676/2020 was parented from unsampled36. These relationships are estimated by the neighbor-joining method using RapidNJ.

**Figure 3:**
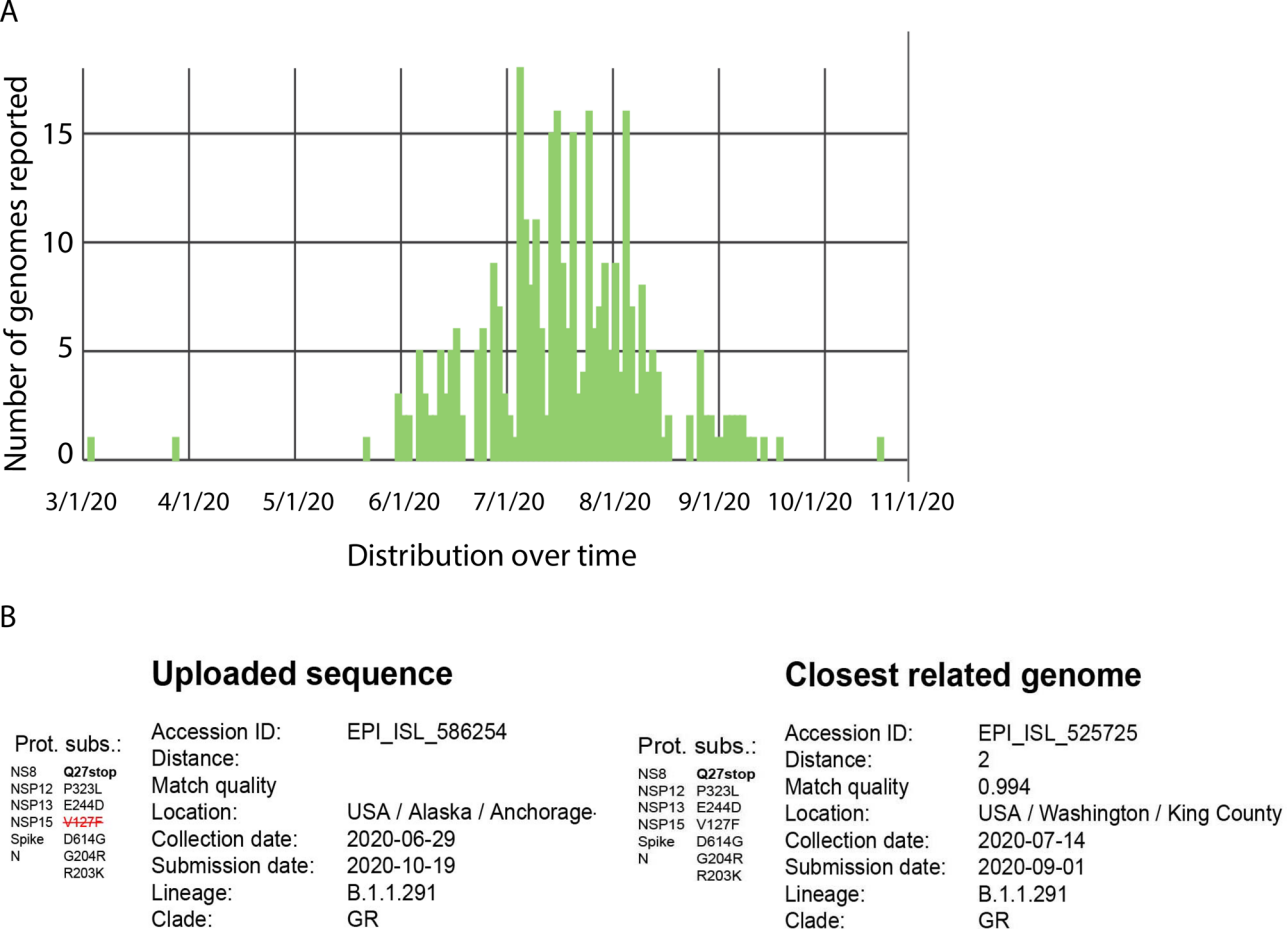
(**A**) Distribution over time of related genomes. A total of 340 related genomes to this novel variant (EPI_ISL_586254) have been published in GISAID with collection dates ranged from 3/6/2020 to 10/21/2020. x axis indicates the collection date, y axis is the number of related genomes published in GISAID. (**B**) The closest related genome is EPI_ISL_525725, which was collected two weeks later on 7/14/2020 in King County, Washington state, USA. A new point mutation (NSP15 V127F) occurred in EPI_ISL_525725 that was not exist in EPI_ISL_586254 (highlighted and crossed in red).

We performed a search using GISAID database in order to find related genomes with a distance of 6 or less from hCoV-19/USA/AK-PHL676/2020. Figure 3 is the summary of GISAID analysis that shows the distribution over time of related genomes. A total of 340 related genomes to this novel variant (EPI_ISL_586254) have been published in GISAID with collection dates ranged from 3/6/2020 to 10/21/2020. As shown in Fig. 3, the closest related genome is EPI_ISL_525725, which was collected two weeks later on 7/14/2020 in King County, Washington state, USA (Fig. 3B). Note there is a new point mutation (NSP15 V127F) in EPI_ISL_525725, which does not exist in hCoV-19/USA/AK-PHL676/2020 (EPI_ISL_586254). Overall, the minimum quality of the matches was 0.95. Amongst the related genomes, the most frequent country was USA (99.4% of genomes), the most frequent lineage was B.1.1.291 (98.8% of genomes), and 84.4% of the related genomes were from samples collected between 05/14/2020 and 06/25/20200.

## Discussion

Understanding the ongoing evolution of SARS-CoV-2 is essential to control and ultimately end the pandemic as the virus continuously mutates and demonstrates adaptive evolution^10^. In this report, we present a SAR-CoV-2 novel variant that contains a truncated ORF8 protein mutation. The wide distribution of this mutation indicates that this truncated ORF8 protein mutation may provide the virus a growth advantage and adaptive evolution.

The effects of ORF8 protein and its functions are still uncertain but a truncated ORF8 could affect antibody response, severity of infection and inflammatory response. This confirmed truncation of 288 nucleotides from the ORF8 region could affect how the human host reacts to this variant of SARS-CoV-2. One study found a 382-nucleotide deletion in the ORF8 gene^11^. They then studied different mutations and deletions in the ORF8 gene and looked at how it would affect ORF8 protein function. It was found that variants with significantly shorter ORF8 proteins had a higher replicative fitness as well as a heightened antibody response^11^. This deletion has been seen in many variants but the effects are not concrete yet. This study compared hospitalized patients with COVID-19 who either had the wild type ORF8 gene or the deletion in the ORF8 gene. They found that development of hypoxia requiring supplemental oxygen was less frequent in the group with the deletion in the ORF8 gene^12^. This could indicate that the truncation in the ORF8 could be associated with milder infections. It has also been suggested that the emergence of ORF8 deletions may be due to immune-driven selection^11^. As this virus continues to replicate in new hosts, we expect to see more mutations in the ORF8 region due to both random mutation and selection.

Our results from identifying the genomic organization of this novel variant and then comparing it to the closely related variants show that it is most likely that this variant came from Washington State. The maximum likelihood phylogenetic tree analysis shows that there are 340 related variants published in GISAID with most of them identified in USA that all have the same truncated ORF8 gene mutation and are most closely related to this Alaskan novel variant.

With SARS-CoV-2 spreading rapidly across the globe, understanding the genome sequence of different variants has become increasingly important. Different studies have looked at the significance of mutations in many SARS-CoV-2 variants. One study did an analysis of 2492 complete genomes and found of those there were 1516 different nucleotide-level variations at different positions^13^. This leads to the understanding that there are many variations in the SARS-CoV-2 genome and an increasing amount as the virus continues to infect more people. The heterogeneity of this virus needs to be better understood when looking at the implications of mutations and how they can affect immune response in humans along with the severity of the disease. Understanding the differences in mutated protein structure is important when trying to create a lasting vaccine that will protect against SARS-CoV-2 as it continues to mutate in the population.

Understanding the genomic makeup of SARS-CoV-2 is essential as we combat the effects of the COVID-19 pandemic. Knowing which variants and what mutations may be circulating in the state of Alaska provides information into how infectious the virus is and how it may be evolving. This study states that ORF8 is a rapidly evolving accessory protein that has been proposed to interfere with immune responses^14^. If we can increase whole genome sequencing in order to gain a better understanding of the genomic makeup of this virus, the next step will be to better understand each protein’s role in the evolution of SARS-CoV-2 and how a mutation could change that role. If we can confirm that a mutation in ORF8 changes the immune response of the virus then we are better equipped to deal with the pandemic.

## Conclusions

Our results show a novel variant of SARS-CoV-2 with a truncated ORF8 gene. This shortens the translated ORF8 protein from 121 to 26 aa. This variant belongs to Pango lineage B.1.1291 but ORF8 truncated mutations are found in other lineages as well. The wide distribution of ORF8 truncated mutations indicate that this mutation may provide the virus a growth advantage and adaptive evolution. 

## Supplementary Information

Supplementary Information.

## Data Availability

The full genomic sequencing data of this SARS-CoV-2 isolate has been submitted to GISAID (virus name: hCoV-19/USA/AK-PHL676/2020; Accession ID: EPI_ISL_586254) and NCBI (GenBank: MW264435.1).
